# Pharmacological inhibition of the PI3K/PTEN/Akt and mTOR signalling pathways limits follicle activation induced by ovarian cryopreservation and in vitro culture

**DOI:** 10.1186/s13048-021-00846-5

**Published:** 2021-07-19

**Authors:** Carmen Terren, Michelle Nisolle, Carine Munaut

**Affiliations:** 1 Laboratory of Tumor and Development Biology, GIGA-Cancer, University of Liège, Tour de Pathologie (B23), Site Sart-Tilman, Building 23/4, Avenue Hippocrate, 13, 4000 Liege, Belgium; 2 Department of Obstetrics and Gynecology, Hôpital de La Citadelle, University of Liège, B-4000 Liège, Belgium

**Keywords:** Fertility preservation, Cryopreservation, Transplantation, Follicular activation, Primordial follicles, Signalling pathways

## Abstract

**Background:**

Cryopreservation and transplantation of ovarian tissue (OTCTP) represent a promising fertility preservation technique for prepubertal patients or for patients requiring urgent oncological management. However, a major obstacle of this technique is follicle loss due to, among others, accelerated recruitment of primordial follicles during the transplantation process, leading to follicular reserve loss in the graft and thereby potentially reducing its lifespan. This study aimed to assess how cryopreservation itself impacts follicle activation.

**Results:**

Western blot analysis of the PI3K/PTEN/Akt and mTOR signalling pathways showed that they were activated in mature or juvenile slow-frozen murine ovaries compared to control fresh ovaries. The use of pharmacological inhibitors of follicle signalling pathways during the cryopreservation process decreased cryopreservation-induced follicle recruitment. The second aim of this study was to use in vitro organotypic culture of cryopreserved ovaries and to test pharmacological inhibitors of the PI3K/PTEN/Akt and mTOR pathways. In vitro organotypic culture-induced activation of the PI3K/PTEN/Akt pathway is counteracted by cryopreservation with rapamycin and in vitro culture in the presence of LY294002. These results were confirmed by follicle density quantifications. Indeed, follicle development is affected by in vitro organotypic culture, and PI3K/PTEN/Akt and mTOR pharmacological inhibitors preserve primordial follicle reserve.

**Conclusions:**

Our findings support the hypothesis that inhibitors of mTOR and PI3K might be an attractive tool to delay primordial follicle activation induced by cryopreservation and culture, thus preserving the ovarian reserve while retaining follicles in a functionally integrated state.

**Supplementary Information:**

The online version contains supplementary material available at 10.1186/s13048-021-00846-5.

## Background

The only alternative available for maintaining the fertility of prepubertal patients or female patients needing urgent therapy for aggressive malignancies and those suffering from hormone-sensitive malignancies is cryopreservation of ovarian cortical tissue containing immature primordial follicles followed by auto-transplantation (OTCTP) [1, 2]. The main advantages of auto-transplantation of cryostored ovarian tissue are the restoration of both the endocrine and fertility functions of the gonads, as well as the ongoing fertility provided by the graft, allowing multiple births [2–4]. To date, more than 130 live births after OTCTP have been reported worldwide, with a live birth rate between 20 and 40% [1, 5–9]. With the increasing number of live births, OTCTP has been proposed to move from experimental studies to open clinical application [10, 11]. However, the main challenge associated with this process is follicular loss immediately after grafting, likely due to slow neovascularization, apoptosis [12–15] and/or massive follicular recruitment, which is also known as follicular burnout [16–19]. Enhancement of graft revascularization by delivering both angiogenic and antiapoptotic factors [20–24] or antioxidants to decrease oxidative stress [25–27] has led to limited improvements in graft outcomes [9]. Recently, the use of adipose tissue-derived stem cells in an experimental mice model of one or two-step transplantation strategy evidenced a real improvement of oxygenation and vascularization of the ovarian tissue in the early post-transplantation period leading to a better follicular survival [28–30]. To improve the lifetime of auto-transplanted fragments and ultimately enhance the pregnancy rate among young patients who are cured of cancer, supplemental studies are clearly required to identify additional strategies to limit follicular burnout.

Under normal physiological conditions, the activation and dormancy of primordial follicles, the non-renewable population that determines the reproductive lifespans of women, are controlled by complex activator and repressor pathways, aiming to keep most follicles in a dormant state [31–33]. Follicle activation signals supplied by the phosphatidylinositol-3-kinase (PI3K)/phosphatase and tensin homologue (PTEN)/Akt and mammalian target of rapamycin (mTOR) pathways, among others, are counterbalanced by signals inhibiting follicle activation through the inhibitory paracrine hormone AMH, which is secreted by growing follicles [34]. After ovarian cryopreservation followed by transplantation, dysregulation in pathways that control follicle dormancy, including upregulation of PI3K/Akt/mTOR and downregulation of AMH, appears to accelerate follicle activation, leaving the ovarian reserve in an unbalanced state [16, 17]. Primordial follicles are more tolerant to the cryopreservation process than mature follicles due to their low metabolic activity [35]. The absence of the inhibitory AMH signal may lead to an imbalance towards activating signals, leading to follicle hyperactivation. Massive and rapid recruitment of primordial follicles can then be observed [16].

The PI3K/PTEN/Akt signalling pathway is the most studied regulatory pathway. This pathway is activated by the binding of growth factors to a receptor tyrosine kinase (RTK), which stimulates PI3K and results in the conversion of phosphatidylinositol [4, 5] bisphosphate (PIP2) to phosphatidylinositol [3–5] triphosphate (PIP3). Then, PIP3 stimulates phosphatidylinositol-dependent kinase 1 (PDK1), leading to Akt activation. Translocation of Akt to the nucleus inhibits the activity of the transcription factor forkhead box O3 (FOXO3). FOXO3, therefore, cannot carry out its function to promote apoptosis and cell cycle arrest and is exported out of the nucleus. PTEN negatively regulates this pathway by dephosphorylating PIP3, resulting in its conversion to PIP2 [36, 37]. Recently, inhibitors of the PI3K/PTEN/Akt pathway, such as AS101, were successfully used to prevent follicle activation occurring during chemotherapy [38]. Other inhibitors, such as LY294002, a powerful competitive PI3K inhibitor [39], induce the arrest of cell growth and have been shown to inhibit the growth of ovarian carcinoma cells in vitro and in vivo [40].

Recently, the mitogen-activated protein kinase (MAPK) signalling pathway was shown to be involved in primordial follicle activation through mTOR complex 1 (mTORC1) signalling [41]. Another prerequisite for maintaining the dormancy of primordial follicles is suppression of mTORC1 activity by the tuberous sclerosis 1 and 2 (Tsc1 and 2) complex [42, 43]. Indeed, mice lacking Tsc1 or Tsc2 genes in oocytes showed spontaneous activation of dormant primordial follicles [42, 43]. Tsc2 can also be phosphorylated and inactivated by Akt, leading to activation of mTORC1 and phosphorylation of ribosomal protein S6 (rps6) [44]. Rapamycin, a specific mTOR inhibitor, is used as an immunosuppressant after organ transplantation and as a drug to prevent revascularization in coronary stents [45]. Inhibition of the activation of primordial follicles of 4‐day‐old rat ovaries in vitro was recently demonstrated using rapamycin [45]. Rapamycin has also been shown to be useful in mice to preserve the ovarian follicle pool and prevent premature ovarian failure [46].

Follicle activation after OTCTP is well documented in both preclinical and clinical studies [47–52]. The transplantation process itself is the major cause of accelerated recruitment of primordial follicles, and the PI3K/PTEN/Akt and mTOR pathways are the main pathways involved in this phenomenon [18, 53, 54]. However, the contribution of the cryopreservation process to follicle activation is not yet well studied. Therefore, our first aim was to determine whether follicle activation occurs directly after cryopreservation of murine ovaries and which signalling pathways are involved in cryopreservation-induced follicle recruitment. The second aim was to use in vitro organotypic culture of whole ovaries to test pharmacological inhibitors of the PI3K/PTEN/Akt and mTOR pathways.

## Materials and methods

### Study design

Activation of PI3K/PTEN/Akt and mTOR signalling pathways in 4–8-weeks old or neonatal murine ovaries cryopreserved and/or cultured on transwell membrane inserts with/without inhibitors (LY294002, a powerful PI3K inhibitor or rapamycin, the specific mTOR inhibitor) were determined by Western Blot and immunofluorescence analyses. Follicles were quantified according to their maturation degree on histological sections.

### Collection and preparation of ovaries

C57Bl/6, Nu/Nu, Rag and FVB/N mice (4 to 8-weeks old) were obtained from Charles River Laboratories (France) or were bred and maintained within the accredited Mouse Facility and Transgenics GIGA platform of the University of Liège (Belgium). Ovaries from 3–7-days old C57Bl/6 pups were also collected. The ovaries were removed through small dorsolateral skin incisions and were placed in a transport solution for slow freezing (SF) composed of Leibovitz L-15 medium (Lonza, Verviers, Belgium) supplemented with 10% Fetal Bovine Serum (FBS; Thermo Fisher Scientific, Gibco, Waltham, Mass., USA). In order to cryopreserve only the ovary, adjacent tissues taken at the time of ovariectomy were removed under a binocular microscope. The Animal Ethics Committee of the University of Liège approved this study (# 1934) and all experiments were performed in accordance with relevant guidelines and regulations.

### Slow freezing (SF) and thawing procedure

Ovaries were placed in cryopreservative medium containing Leibovitz L-15 medium supplemented with 10% FBS, 10% dimethylsulfoxide (DMSO; Merck, Darmstadt, Germany) and 0.1 M sucrose. After equilibration in cryopreservation medium for 30 min at 4 °C, ovaries were placed in cryovial tubes (Simport, Montreal, Quebec, Canada) and subsequently cooled in a programmable freezer (CL-8800i System; CryoLogic, Mulgrave, Victoria, Australia) as described previously [55] and stored in liquid nitrogen. For slow freezing with inhibitors, LY294002 (25 µM, InvivoGen, Toulouse, France) or Rapamycin (1 µM, InvivoGen, Toulouse, France) were added in the transport and cryopreservation media.

For thawing, cryovials were incubated at room temperature for 2 min and thawed by rapid immersion at 37 °C in a water bath. To remove cryoprotectants, ovaries were washed three times for 5 min at 37 °C in Leibovitz L-15 medium.

### In vitro culture

After thawing, whole ovaries were cultured for 24 h (except otherwise mentioned) at 37 °C in 12-well plates on tissue culture inserts (ThinCerts 0.4 µm PET; Greiner Bio-One, Kremsmünster, Austria). Culture medium was composed of Dulbecco’s Modified Eagle Medium (Thermo Fisher Scientific, Gibco, Waltham, MA, USA) supplemented with 1% Bovine Serum Albumin (Thermo Fisher Scientific Gibco, Waltham, MA, USA), 1% L-Glutamine (Thermo Fisher Scientific, Gibco, Waltham, MA, USA), 1% of a mixture of insulin (1000 mg/l), transferrin (550 mg/l) and selenium (0.67 mg/l) (Thermo Fisher Scientific, Gibco, Waltham, MA, USA), 1% penicillin–streptomycin (Thermo Fisher Scientific Gibco, Waltham, MA, USA), 0.5% ascorbic acid (Sigma-Aldrich, St. Louis, MO, USA) and 2.5% human FSH (Sigma-Aldrich, St. Louis, MO, USA), supplemented or not with follicle pathway inhibitors (LY294002, 25 µM or rapamycin, 1 µM). When the culture is carried out for more than 2 days, half of the medium was renewed each second day.

### Histological assessment

Ovaries fixed in 4% formaldehyde were paraffin-embedded and serially sectioned (5 µm sections).

Labelling of the LIM-homeobox protein 8 (Lhx8) transcription factor as well as DEAD-box helicase 4 (DDX4) were performed for differential follicle counts (see Additional file 1). In order to assess the activation of pathways involved in follicle activation, labelling of phospho-Akt and phospho-rps6 antigens was carried out. Briefly, sections were deparaffinised and rehydrated, and endogenous peroxidase activity was blocked by incubating the sections in 3% hydrogen peroxide for 20 min at room temperature (RT). Nonspecific binding sites were blocked by incubation in the “Free Blocking Solution” (Cell Signaling, Danvers, USA) for 1 h at RT. Primary antibodies were diluted at different concentrations in the “REAL antibody diluent” (Dako, Glostrup, Denmark) and incubated for one 1 h at RT (rabbit polyclonal anti-Lhx8 (Abcam ab41519, Cambridge, UK) 1/100; rabbit polyclonal anti-DDX4 (Abcam ab13840, Cambridge, UK) 1/600; rabbit monoclonal anti-phospho-Akt (Ser473—Abcam ab81283, Cambridge, UK) 1/250 and rabbit polyclonal anti-phospho-rps6 (Cell Signaling #2211, Danvers, USA) 1/400), followed by incubation with the secondary antibody HRP linked (ENVISION/HRP ready to use, Dako, Glostrup, Denmark) for 30 min at RT. The reaction was either revealed using DAB + (Dako, Glostrup, Denmark) and the sections were counterstained with haematoxylin or the fluorescein tyramide kit (PerkinElmer, Waltham, MA, USA) was used during 10 min for fluorescein staining and sections were mounted with DAPI FluoromountG mounting medium (SouthernBiotech, Birmingham, Alabama, USA).

### Quantification of immunostaining

Follicle pathway activation was determined by computer-assisted image analysis on p-Akt or p-rps6 labelled sections (5 ovaries were analysed per group). Using Photoshop CS4 software (Adobe Systems Incorporated, San Jose, CA, USA), a mask was created to delineate the whole ovarian section to be analysed. The number of cells labelled per mm^2^ was then quantified using MATLAB software (MathWorks, Inc.) as previously described [56].

### Quantification of follicles

The scanned Lhx8-labelled sections were visualized using NDP view software (NDP.view2 Viewing software U12388-01, Hamamatsu Photonics K.K., Japan). Sections were analyzed by light microscopy for the presence of primordial, primary and secondary or more mature follicles based on morphological classification of mouse follicles [57]. The follicular densities (number/mm^2^) were calculated after manually outlining the ovarian surface (NDP view software) and results are expressed in percentage of primordial, primary and secondary or more mature follicles relative to the total number of follicles present in one ovarian slide. Two slides per ovary were analysed blinded by two independent observers.

For quantification of primordial follicles labelled or not by p-Akt or p-rps6, a double immunostaining was performed with DDX4 and p-Akt or p-rps6. Primordial follicles identified by DDX4 staining were manually counted and classified into labelled or non-labelled group. Quantification was performed on at least three ovarian slides for each ovary (*n* = 5 ovaries per group).

### Western blot analysis

For 4–8-weeks old ovaries, three ovaries were pooled by condition to obtain a sufficient protein concentration, whereas for 3–7-days old ovaries, 6–8 ovaries were pooled. The protein extraction was carried out with radio immunoprecipitation assay (RIPA) buffer containing 4% of a protease and phosphatase inhibitor (Roche, Basel, Switzerland). Lysate were collected and protein concentrations determined using a protein assay kit (Bio-Rad Laboratories, Hercules, CA, USA). Equal amounts of protein were denatured and separated by electrophoresis on SDS–polyacrylamide gels. Proteins were then transferred onto a polyvinylidene difluoride membrane (PerkinElmer, Waltham, MA, USA) at 100 V for 1 h. After blocking in a 5% BSA solution, proteins were incubated with respective primary antibodies (Cell Signaling, Danvers, USA), at a dilution of 1/1000, in the blocking solution, overnight at 4 °C. Primary antibodies used were anti-total Akt (#9272) and anti-phospho-Akt (Ser473 #9271), and anti-total-rps6 (#2217) and anti-phospho-rps6 (#2211). The appropriate horseradish peroxidase-conjugated secondary antibody was added to the membrane followed by a 1 h incubation at RT. After sequential washing of the membranes to remove excess secondary antibody, signals were detected using an enhanced chemiluminescence (ECL) kit (PerkinElmer, Waltham, MA, USA) according to the manufacturer’s instructions in a LAS4000 imager (Fujifilm, Tokyo, Japan). Protein levels were quantified using QuantityOne Analysis software. Data are represented as the ratio of phosphorylated proteins to total proteins and are expressed as the fold-change compared to control group. β-actin expression was measured to verify equal loading.

### Statistical analysis

Statistical analyses were performed using GraphPad Prism software (GraphPad, San Diego, CA, USA), using a Mann–Whitney test for comparisons between two groups. All data are presented as means ± SEM. A probability of *p* < 0.05 was considered to be statistically significant.

## Results

### Slow freezing-induced follicle activation through the PI3K/PTEN/Akt and mTOR signalling pathways is reduced by the addition of LY294002 or rapamycin

**Fig. 1:**
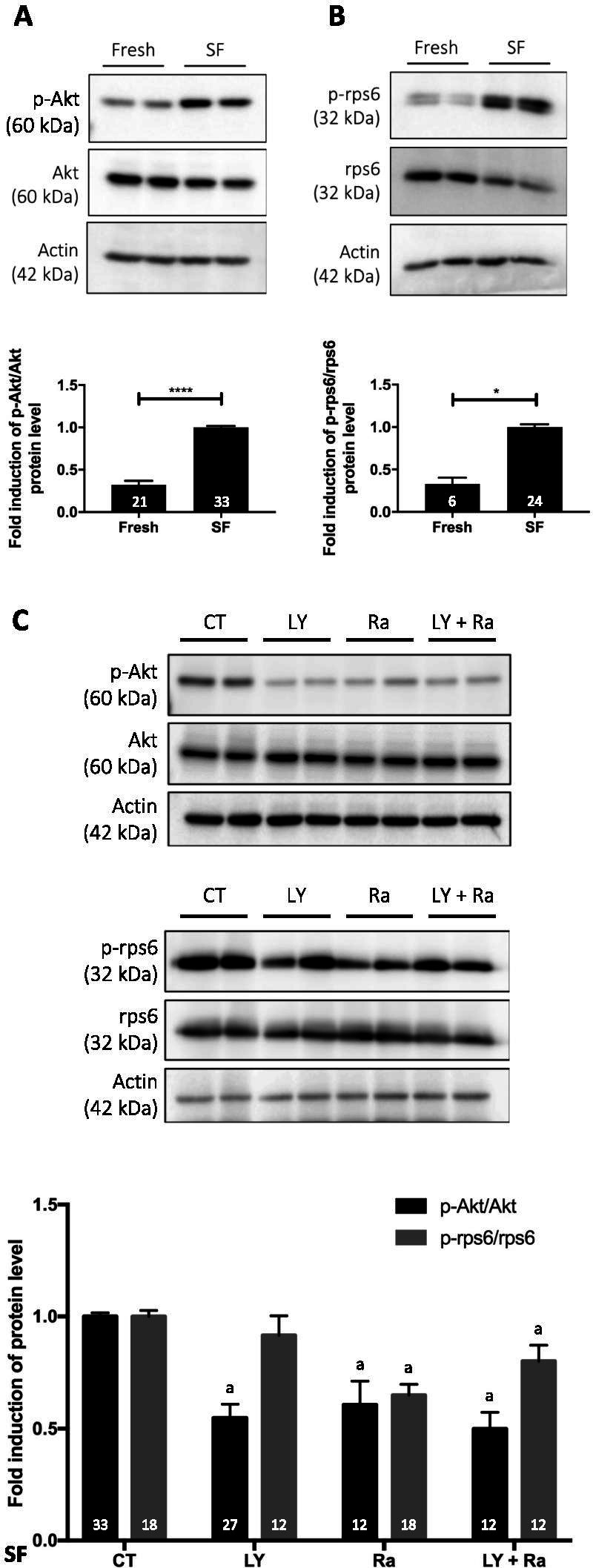
Effect of cryopreservation and the addition of pharmacological inhibitors of the PI3K/PTEN/Akt and mTOR signalling pathways during cryopreservation on follicle activation. Ratio of phosphorylated to total protein forms of Akt (**A**) and rps6 (**B**). **C** Addition of inhibitors to cryopreservation media. SF = slow-frozen/thawed ovaries with or without inhibitors: CT = control; LY = LY294002; Ra = rapamycin. Ovaries from C57Bl/6 and Nu/Nu mice, 4 weeks old. a significant difference compared to the corresponding CT. Numbers in columns represent the number of ovaries analysed per group. 3–5 experimental replicates were performed. *p ≤ 0.05, ****p ≤ 0.0001

The impact of the cryopreservation process on subsequent follicle activation was evaluated in cryopreserved-thawed murine ovaries compared to fresh ovaries (4 weeks old ovaries). By Western blot analysis, the PI3K/PTEN/Akt and mTOR signalling pathways were found to be activated in frozen/thawed ovaries (Fig. 1A-B).

In order to limit follicle activation during cryopreservation, pharmacological inhibitors of the activated signalling pathways were added to the media during ovary preparation and cryopreservation. The addition of LY294002, a PI3K inhibitor, effectively decreased the ratio of phosphorylated Akt to total Akt (p-Akt/Akt) compared to cryopreserved ovaries in control medium (Fig. 1C). LY294002 is a specific inhibitor of PI3K, and the mTOR pathway was not affected by this inhibitor. The addition of rapamycin, a specific mTOR inhibitor, reduced both Akt and mTOR activation. No additional or synergistic effect was observed when ovaries were cryopreserved in the presence of both LY294002 and rapamycin (Fig. 1C).

### In vitro organotypic culture-induced activation of the PI3K/PTEN/Akt pathway is counteracted by cryopreservation with rapamycin and in vitro culture in the presence of LY294002

**Fig. 2:**
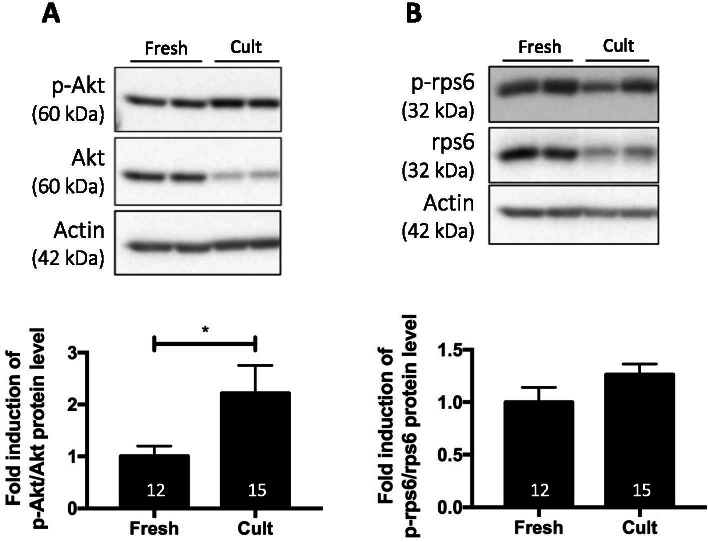
Effect of in vitro organotypic culture on follicle activation. Ratio of phosphorylated to total protein forms of Akt (**A**) and rps6 (**B**). Cult = organotypic culture for 24 h. Ovaries from Nu/Nu mice, 8 weeks old. Numbers in columns represent the number of ovaries analysed per group. 2 experimental replicates were performed. *p ≤ 0.05

In vitro culture is a useful tool to study follicle activation since follicles are activated throughout the culture period [58–61]. An activation of the PI3K/PTEN/Akt signalling pathway was confirmed in fresh whole ovaries (8 weeks old) maintained in organotypic culture for 24 h compared to fresh non-cultured ovaries (Fig. 2A). Only minimal and non-significant activation of the mTOR pathway was observed (Fig. 2B).

**Fig. 3:**
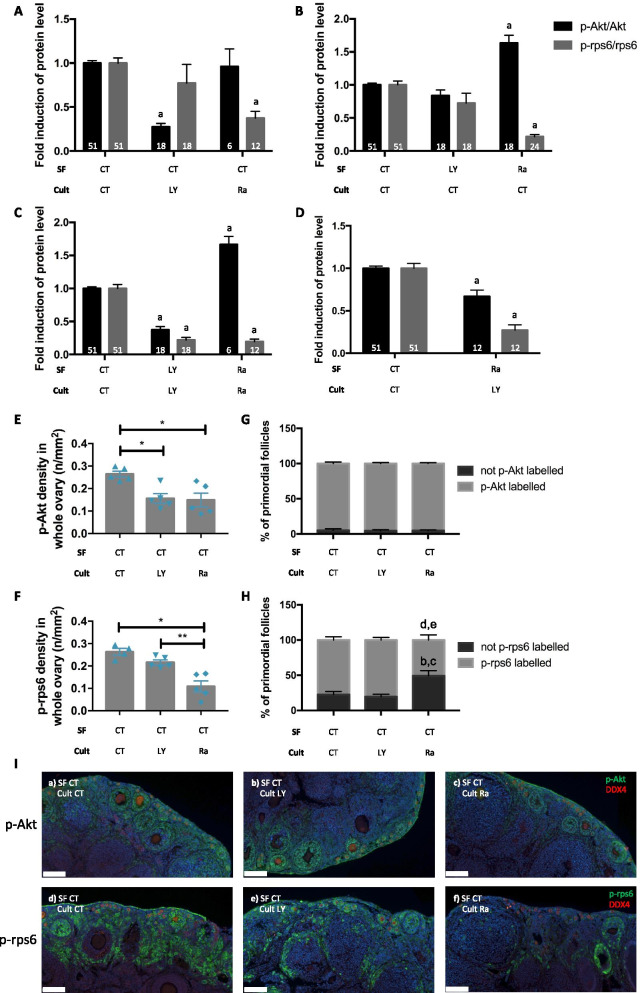
Effect of the addition of pharmacological inhibitors of the PI3K/PTEN/Akt and mTOR signalling pathways during cryopreservation and/or in vitro organotypic culture on follicle activation. Ratio of phosphorylated to total Akt and rps6 for **A** ovaries cryopreserved without inhibitors and cultured for 24 h with LY or Ra; **B** ovaries cryopreserved with LY or Ra and cultured for 24 h without inhibitors; **C** ovaries cryopreserved and cultured for 24 h with inhibitors or **D** ovaries cryopreserved with Ra and cultured for 24 h with LY. **E** and **F** Computer-assisted quantification of the immunofluorescence staining density of phosphorylated forms of Akt and rps6 in the whole ovarian section. **G** and **H** Quantification of primordial follicles labelled or not by p-Akt or p-rps6. **I** Representative images of p-Akt (a-c) and p-rps6 (d-f) staining for the different groups. Red staining = DDX4; green staining = p-Akt or p-rps6. SF = slow-frozen/thawed ovaries with or without inhibitors followed by organotypic culture (cult) for 24 h with or without inhibitors. CT = control; LY = LY294002; Ra = rapamycin. a significant difference compared to the corresponding control; b significant difference compared to control not labelled primordial follicles. c significant difference compared to not labelled primordial follicles in ovaries cultured with LY294002; d significant difference compared to control labelled primordial follicles; e significant difference compared to labelled primordial follicles in ovaries cultured with LY294002. Ovaries from C57Bl/6, Nu/Nu and Rag mice, 4–8 weeks old. Numbers in columns represent the number of ovaries analysed per group. **E**-**I ***n* = 4–5 ovaries per group. 2–3 experimental replicates were performed. *p ≤ 0.05, **p ≤ 0.01. Scale bar represents 100 µm

Combination of cryopreservation and culture (24 h) both with or without inhibitors was performed to evaluate the effects of signalling pathway inhibitor treatment during in vitro organotypic culture after cryopreservation. When ovaries were cryopreserved without inhibitors and afterwards cultured for 24 h with inhibitors, a decreased activation of the PI3K/PTEN/Akt pathway was observed with the addition of LY294002 in the culture medium (Fig. 3A). Likewise, the presence of rapamycin in culture reduced the activation of the mTOR pathway.

The presence of those pharmacological inhibitors during the cryopreservation process itself followed by 24 h of culture without inhibitors was evaluated (Fig. 3B). Cryopreservation in presence of LY294002 had no effect on the PI3K/PTEN/Akt and mTOR pathways. The addition of rapamycin during cryopreservation effectively inhibited the mTOR pathway and a significant increase of the PI3K/PTEN/Akt was observed (Fig. 3B).

We next analysed the addition of the inhibitors both during cryopreservation and in vitro culture (Fig. 3C). The presence of LY294002 in the freezing and culture media had an inhibitory effect on both pathways and the addition of rapamycin reduced the activation of the mTOR pathway, while increased activation of the PI3K/PTEN/Akt was observed compared to the control condition (Fig. 3C).

From these results, we concluded that the addition of LY294002 during in vitro culture effectively reduced activation of the PI3K/PTEN/Akt pathway, whereas the presence of rapamycin during cryopreservation successfully inhibited the mTOR pathway. Therefore, we performed a mixed experiment: ovaries were cryopreserved in the presence of rapamycin, and LY294002 was added for 24 h in the in vitro culture (Fig. 3D). With this combination, the PI3K/PTEN/Akt and mTOR pathways were both inhibited compared to control ovaries (cryopreserved and cultured in control medium) (Fig. 3D).

To further verify the involvement of these signalling pathways during cryopreservation and in vitro culture, the distribution of components of Akt and mTOR in the whole ovarian section was quantified by computer-assisted detection of the immunofluorescence staining density. Phosphorylated forms of Akt (p-Akt) and rps6 (p-rps6) were used to evaluate the inhibitory effects of treatments on the PI3K/PTEN/Akt and mTOR pathways. Indeed, when ovaries were cryopreserved without inhibitors followed by in vitro culture for 24 h with LY294002 or rapamycin, the staining density of p-Akt and p-rps6 in the whole ovarian section was decreased compared to culture without inhibitors (Fig. 3E–F and I). Since the aim of this study is to analyse follicle activation after cryopreservation and organotypic culture, a restriction of the immunostaining of p-Akt and p-rps6 only to primordial follicles was performed using a double immunostaining of DDX4 and p-Akt or DDX4 and p-rps6. The percentage of primordial follicles labelled by p-Akt is similar in all groups. However, when cryopreserved ovaries were cultured with rapamycin for 24 h, the percentage of primordial follicles stained by p-rps6 decreased compared to cryopreserved ovaries cultured without inhibitors or with LY294002. Concomitantly, the percentage of non-labelled by p-rps6 primordial follicles was higher in cryopreserved ovaries cultured with rapamycin compared to ovaries cultured in a control medium or with LY294002 (Fig. 3G-I).

### Follicle development is affected by in vitro organotypic culture, and PI3K/PTEN/Akt and mTOR pharmacological inhibitors preserve primordial follicle reserve

Fresh and SF ovaries were cultured for 2 or 4 days, and follicles were quantified according to their maturation. In order to facilitate counting, oocytes were labelled by Lhx8 immunostaining (see Additional file 1). The percentage of primordial follicles relative to total number of follicles in the analysed ovarian section decreased in cultures of fresh and SF ovaries with increasing culture time compared to non-cultured ovaries. No significant change in primary follicle percentage was found for fresh ovaries, however it decreased in SF ovaries cultured for 2 or 4 days compared to SF non-cultured ovaries. The percentage of secondary or more mature follicles increased with increasing culture time both in fresh and SF ovaries (Table 1). Furthermore, primordial follicle percentage of SF ovaries cultured for 2 days significantly decreased compared to fresh ovaries cultured for the same time accompanied by an increase in secondary or more mature follicle percentage.

**Table 1:** Mean number of follicles after in vitro organotypic culture of fresh or cryopreserved whole ovaries

	**Fresh**			**SF**		
	**w/o cult**	**Cult 2 days**	**Cult 4 days**	**w/o cult**	**Cult 2 days**	**Cult 4 days**
**Primordial**	28,94 ± 2,614	22,99 ± 1,853	14,62 ± 1,958^a,c^	22,4 ± 1,175	14,78 ± 1,587^b,c^	11,13 ± 1,29^b^
**Primary**	17,68 ± 2,068	15,26 ± 1,541	16,95 ± 2,471	18,04 ± 1,026	11,16 ± 1,219^b^	12,48 ± 1,155^b^
**Secondary or more**	53,37 ± 3,503	61,75 ± 2,48	68,44 ± 3,757^a^	59,56 ± 1,798	74,05 ± 2,346^b,c^	76,39 ± 2,163^b^

Fresh or SF (slow frozen/thawed) ovaries from FVB/N mice, 7-weeks old, *n* = 6–9 ovaries per group. w/o cult = without culture

Results are expressed in percentage of primordial, primary and secondary or more mature follicles relative to the total number of follicles present in one ovarian slide. Mean ± SEM

^a^different from Fresh w/o cult, ^b^different from SF w/o cult, ^c^different from Fresh cult 2 days

Preservation of primordial follicles was observed when rapamycin was added to the culture medium for a culture time of 24 h compared to the control condition. Indeed, more primordial follicles were counted in the ovarian sections cultured with rapamycin compared to an ovary cultured without inhibitor. No difference in primary follicle percentage was observed, and the percentage of secondary or more mature follicles trend to be lower with rapamycin after a 24 h culture period compared to the control (Table 2).

**Table 2:** Mean number of follicles after in vitro organotypic culture of cryopreserved whole ovaries in presence of pharmacological inhibitors

	**CT**	**LY**	**Ra**
**24 h**			
**Primordial**	20.53 ± 1.906	21.22 ± 1.584^b^	26.81 ± 1.516^a^
**Primary**	16.61 ± 3.265	8.993 ± 2.302	7.72 ± 1.348
**Secondary or more**	42.37 ± 2.56	48.65 ± 3.192^b^	38.73 ± 2.397

Ovaries from Nu/Nu mice, 4-weeks old, *n* = 5 ovaries per group

Results are expressed in percentage of primordial, primary and secondary or more mature follicles relative to the total number of follicles present in one ovarian slide. Mean ± SEM

*CT* Control, *LY* LY294002, *Ra* Rapamycin

^a^different from CT, ^b^different from Ra

### The addition of rapamycin during cryopreservation and LY294002 during in vitro culture of neonatal ovaries is effective in reducing follicle activation

**Fig. 4:**
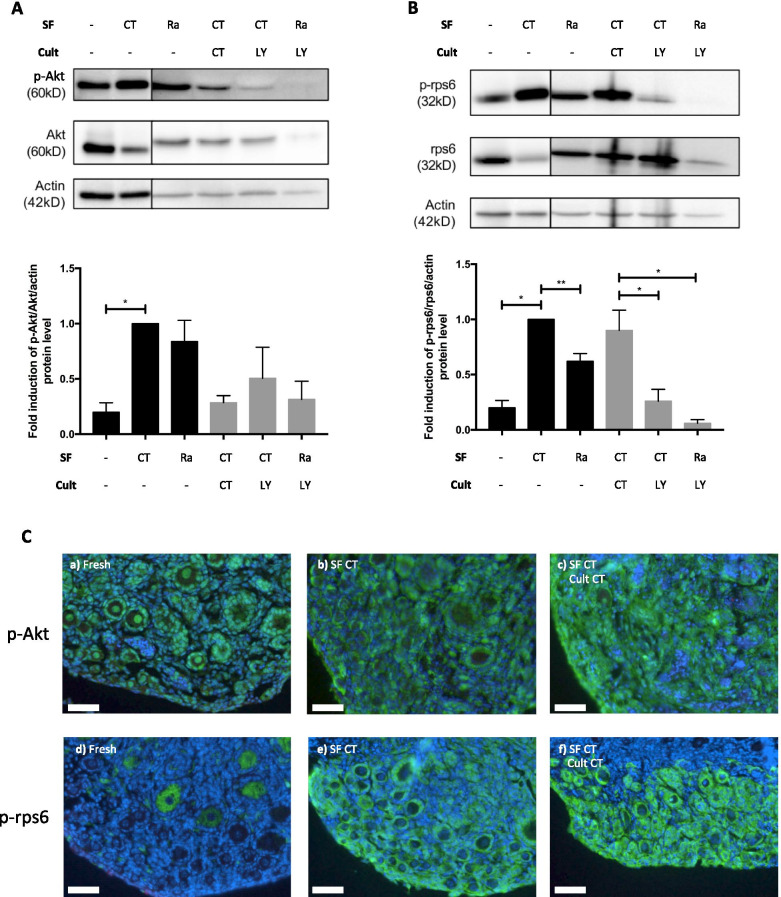
Effect of the addition of pharmacological inhibitors of PI3K/PTEN/Akt and mTOR signalling pathways during cryopreservation and/or in vitro organotypic culture on follicle activation of neonatal ovaries. **A** and **B** Ratio of phosphorylated to total protein Akt and rps6. **C** Representative images of p-Akt (a-c) and p-rps6 (d-f) staining for the different groups. SF = slow-frozen/thawed ovaries with or without inhibitors followed or not by organotypic culture (cult) of 24 h with or without inhibitors. CT = control; LY = LY294002; Ra = rapamycin. Scale bar represents 50 µm. 3–5 experimental replicates were performed. Ovaries from C57Bl/6 mice, 3–7 days old; *n* = 24–40 ovaries per group

To assess the efficacy of pharmacological inhibitors on ovaries containing a larger pool of primordial follicles, 3–7-postnatal-day ovaries were used. These ovaries were cryopreserved and cultured for 24 h in the presence or not with LY294002 or rapamycin. Western blot analysis of these neonatal ovaries revealed that the PI3K/PTEN/Akt and mTOR pathways were activated by cryopreservation (Fig. 4A-B). This activation was inhibited by cryopreservation in the presence of rapamycin. Only a limited inhibitory effect of LY294002 in culture medium on the PI3K/PTEN/Akt pathway was observed, but LY294002 in culture effectively inhibited the mTOR pathway and did so even more when it was combined with rapamycin in transport and freezing media (Fig. 4A and B).

Histological assessments revealed that p-Akt staining is present in fresh ovaries. Furthermore, activation of the PI3K/PTEN/Akt and mTOR pathways after cryopreservation of young murine ovaries compared to fresh ovaries was observed. These pathways were also activated by a 24-h in vitro culture (control medium) (Fig. 4C).

## Discussion

A major obstacle of OTCTP is follicle loss due to, among other factors, accelerated recruitment of primordial follicles during the transplantation process [18, 53, 54]. Our first aim was to assess how cryopreservation itself impacts follicle activation. Our results showed that the PI3K/PTEN/Akt and mTOR signalling pathways were activated by slow freezing. These observations led us to consider adding specific inhibitors during the cryopreservation process, in both the transport and freezing media. Our results indicate that LY294002, a competitive PI3K inhibitor, inhibited the PI3K/PTEN/Akt pathway but not the mTOR pathway. The contact time of the metabolically active ovary with the inhibitor LY294002 during the freezing process could be too short to inhibit the mTOR pathway, since this inhibition would be indirect [44]. Rapamycin, a specific mTOR inhibitor, reduced the activation of mTOR and the PI3K/PTEN/Akt pathway. Combining LY294002 and rapamycin at the same time result in  no additive effect. To the best of our knowledge, this strategy regarding the addition of pharmacological signalling pathway inhibitors during slow freezing has not yet been assessed. Only two studies evaluated inhibitors during cryopreservation (vitrification). One of these is the study by Kong et al., which evaluated whether exogenous supplementation of AMH (a physiological inhibitor of follicle activation) during vitrification and warming could preserve primordial follicles. In this study, the addition of AMH was not effective [62]. Liu et al. tested recently a new vitrification protocol with pre-treatment of rapamycin. The rapamycin treatment has been shown to inhibit the activation of mTOR signalling pathway in ovaries analysed directly after thawing or in ovaries shortly grafted in the recipient mice [63].

Our next aim was to use whole ovary organotypic culture as a model to study in vitro follicle activation pathways. In vitro culture is a useful tool to study follicle activation since follicles are activated throughout the culture period [58–61]. Among these pathways tested, only the PI3K/PTEN/Akt pathway showed an activation after 24 h of in vitro culture.

Pathway activation was also studied in ovaries undergoing both processes, namely, slow freezing and subsequent in vitro culture, both with and without inhibitors. Our results showed that the inhibitors LY294002 and rapamycin were able to act on both PI3K/PTEN/Akt and mTOR pathways, indicating the interaction between these pathways. Indeed, the mTOR pathway is located downstream of the PI3K/PTEN/Akt pathway; Akt can interact with Tsc1/Tsc2. Thus, inhibition of the mTOR pathway is expected when PI3K is inhibited by LY294002 [44].

Western blot and immunofluorescence analyses showed that in vitro incubation with LY294002 or rapamycin significantly inhibited the activation of the PI3K/PTEN/Akt or mTOR pathway, respectively. Similar results were previously observed by Hu et al. using another Akt inhibitor, MK2206, which inhibited primordial follicle activation in mouse postnatal-day-3 ovaries cultured for 8 days [64]. Reduced p-Akt levels in follicles were also observed by immunohistochemistry in a recent in vitro study of ovine ovarian tissue cultured for 7 days in combination with epigallocatechin-3-gallate (EGCG), which has strong antioxidant and anti-apoptotic properties and activates the PI3K/PTEN/Akt pathway [65]. Our results are in agreement with the recent study by Xie et al. (2020), showing that rapamycin inhibited the activation of primordial follicles of 4‐day‐old rat ovaries in vitro [45].

Quantifications of immunostaining of p-Akt and p-rps6 were performed by taking into consideration the whole ovarian section but also by restricting the analyses to primordial follicles. Indeed, primordial follicles stained by p-Akt or p-rps6 were counted thanks to a double immunostaining of DDX4 and p-Akt or DDX4 and p-rps6. No difference in primordial follicle percentage labelled for p-Akt was found between cryopreserved ovaries cultured in a control medium compared to cultures with LY294002 or rapamycin. However, when p-Akt staining in the whole ovarian section was considered, a decreased staining density was observed in cryopreserved ovaries cultured with inhibitors compared to the control condition. This difference could be explained by the fact that stromal and follicle cells are communicating to trigger follicle activation [66]. The reduced staining density of p-Akt in the whole ovarian section is therefore probably related to stromal cells. The effects of LY294002 and rapamycin on follicle activation could be indirect. Inhibitors are probably acting on primordial follicles though a dialogue between stromal and follicle cells. For the mTOR pathway, a reduced staining density of p-rps6 in the whole ovarian section for cryopreserved ovaries cultured with rapamycin is associated with a reduced number of p-rps6-stained primordial follicles as compared to the control condition and ovaries cultured with LY294002. The addition of rapamycin during the ovarian cryopreservation process followed by in vitro culture with LY294002 effectively inhibited both the mTOR and PI3K/PTEN/Akt pathways. The next step would be to test this combination in vivo. Ideally, ovaries should be cryopreserved with rapamycin and transplanted in mice and then locally or systematically treated with LY294002 to limit follicle hyperactivation linked to the transplantation process.

When ovaries were cryopreserved in presence of rapamycin and cultured in control medium or with rapamycin, an increased ratio of p-Akt/Akt was observed. This increase could be due to the feedback activation of Akt after mTOR inhibition [67]. In cancers, the activation of PI3K/Akt kinase is a common event and it is known that these kinases are hypersensitive to mTOR inhibitors, including rapamycin [68, 69]. Therefore, by analogy, the suppression of mTOR function and prevention of Akt activation, by a combination of mTOR and PI3K/PTEN/Akt pathway inhibitors, could limit early follicle activation after cryopreservation and organotypic in vitro culture.

Follicle density analyses revealed that follicle activation occurs during culture of both fresh and SF ovaries. Indeed, a lower percentage of primordial follicles and a higher percentage of growing follicles were found in fresh and SF ovaries cultured for 2 or 4 days compared to control non-cultured ovaries. Follicle activation was also observed in SF ovaries cultured for 2 days compared to fresh ovaries cultured for the same culture time. Cryopreserved ovaries cultured for 24 h in the presence of rapamycin showed a higher percentage of primordial follicles relative to the total number of follicles in the ovarian slide. No significant difference in the percentage of primary follicles after 24 h of culture, whether with LY294002 or rapamycin, was noted. A trend towards a lower proportion of follicles that reached the secondary stage was observed in cultured ovaries with rapamycin compared to ovaries cultured without inhibitors, indicating a lower rate of activation and transition of primordial follicles into mature follicles.

In our study, different strains of mice were included, from C57Bl/6, FVB/N to immunodeficient Nu/Nu and Rag mice. It could be argued that different mice strains could not contain the same number of ovarian follicles [70–72]. According to Pepling et al., different numbers of follicles in mouse ovaries could be due to methods used to assess their number, leading to differences between animals of the same age and genetic strain [73]. More recently, differences in primordial follicle population among different mice strains was demonstrated to be attenuated by different patterns of follicular recruitment and atresia [74]. Nevertheless, in our study, each experiment was performed with its own mice strain control and altogether the efficiency of inhibitors such as LY294002 and rapamycin was confirmed to limit follicular activation either by in vitro analysis of signalling pathways or by immunohistochemistry and follicular counting both in mature and juvenile ovaries.

All results could therefore be considered strong and could also mimic the human situation. Indeed, women have also different follicular reserves which decline through time more or less rapidly to finally reach the menopausal status between the age of 40–60 [75, 76]. Furthermore, for most applications, the use of robust and diverse subjects is preferred, so that conclusions obtained will be maximally generalizable across conditions and populations [77].

Most of our experiments were performed with ovaries from 4- to 8-week-old mice. These ovaries are equivalent to mature ovaries in which the stock of primordial follicles is already diminished. We confirmed the most striking results with juvenile ovaries (3–7 days old). In clinical setting, OTCTP is a process mainly for prepubertal patients and it is therefore relevant to study follicle activation both in juvenile ovaries and in older ones. These neonatal murine ovaries contain a large reserve of primordial follicles, and freezing- and culture-induced follicle activation is therefore more pronounced. Indeed, Western blot and immunofluorescence analyses revealed activation of the PI3K/PTEN/Akt and mTOR pathways by cryopreservation.

Furthermore, since activation is more pronounced in juvenile ovaries, we performed experiments with pharmacological inhibitors with these ovaries. Transport and freezing media were supplemented with rapamycin, and decreased activation of both the PI3K/PTEN/Akt and mTOR pathways was observed. Since the results of combining rapamycin during cryopreservation and LY294002 during the culture period with 4- to 8-week-old ovaries were promising, the same combination was tested with neonatal ovaries. Decreased activation of the mTOR signalling pathways was detected. Moreover, immunofluorescence analyses of neonatal ovaries showed p-Akt staining but only minimal p-rps6 staining in fresh ovaries. However, the staining densities of both increased when ovaries were cryopreserved and/or cultured in control medium for 24 h. These results are in accordance with the results of Masciangelo et al. (2019), who demonstrated that mTOR phosphorylation is a late event in the signalling cascade, occurring when follicles have already started to grow and when further growth is needed [17].

One of the limitations of our study is that no tissue/cell survival tests after LY294002 or rapamycin treatment were performed. Nevertheless, these inhibitors have already been used in several in vitro and in vivo studies [41, 63, 78–82]. Another limit is that murine ovaries were used for this in vitro study. To fully document the efficiency of LY294002 and rapamycin to limit cryopreservation and transplantation-induced follicle recruitment, these inhibitors should be tested in an in vivo model.

## Conclusion

**Fig. 5:**
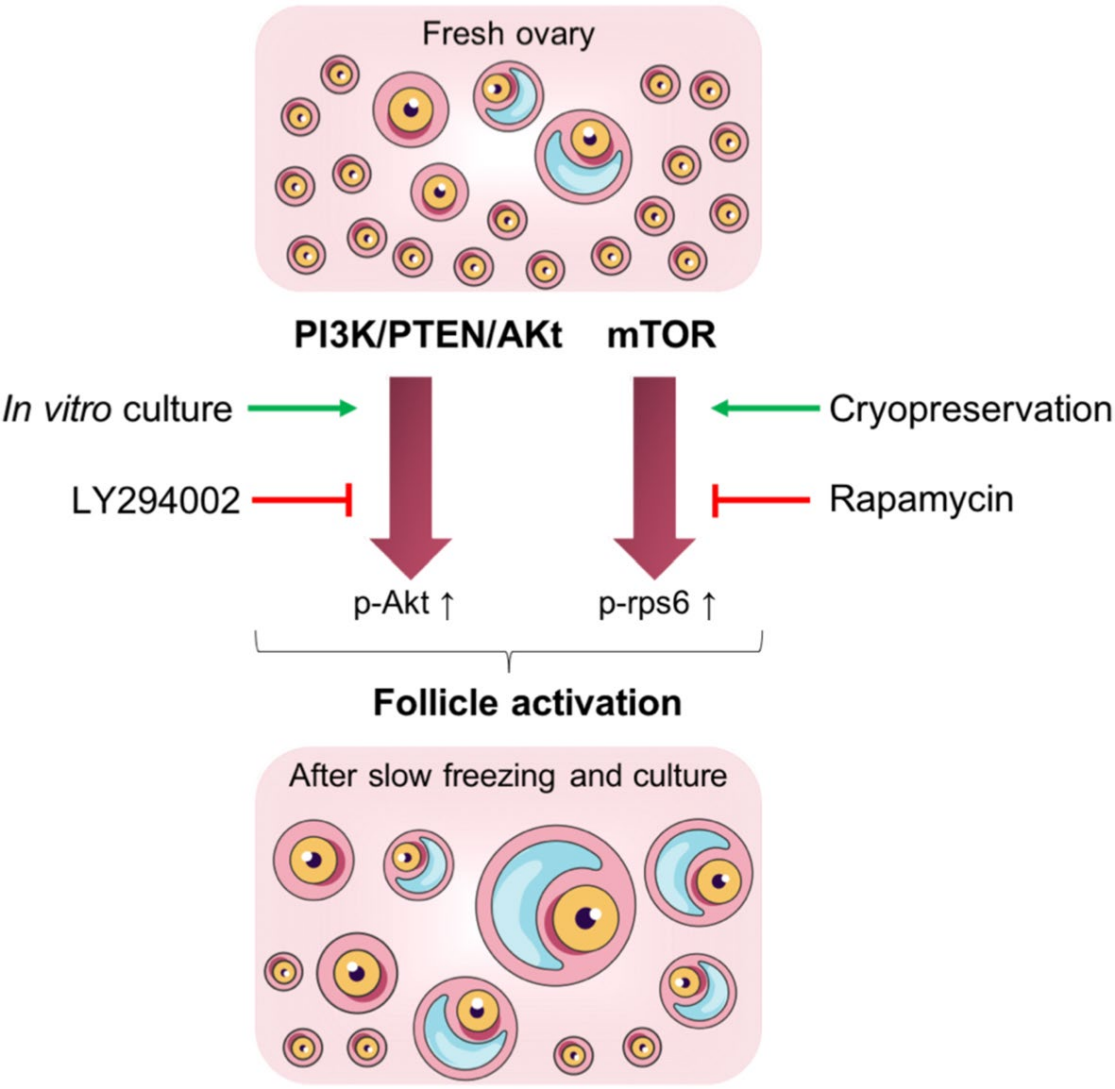
General overview of follicle activation induced by cryopreservation and in vitro culture. PI3K/PTEN/Akt and mTOR pathways are implicated in follicle activation in murine ovaries after cryopreservation and/or in vitro culture via an increase of p-Akt and p-rps6. LY294002 and rapamycin can limit this follicle activation

In conclusion, our results could open up new approaches for preserving follicular reserve in the OTCTP process through the use of pharmacological inhibitors of the PI3K/PTEN/Akt and mTOR signalling pathways. Indeed, the slow freezing process is responsible for follicle pathway activation, and this activation can be reduced by the use of the PI3K inhibitor LY294002 and the mTOR inhibitor rapamycin. These inhibitors are also effective in inhibiting cell culture-induced activation. Our findings provide support for the hypothesis that mTOR and PI3K inhibitors might represent an attractive tool to delay cryopreservation- and culture-induced primordial follicle activation, therefore protecting the ovarian reserve while maintaining follicles in a functionally integrated state (Fig. 5). However, in vivo studies on mouse ovaries are necessary to confirm their in vivo efficiency. For ethical reasons, we used mouse ovaries, and our results will need to be confirmed with human samples (in vitro and in vivo).

## Supplementary Information

**Additional file 1.** Representative images of LIM-homeobox protein 8 (Lhx8) immunostaining. Ovaries from Nu/Nu mice, 4 weeks old, (A) cultured for 24 h in control medium or (B) cultured for 24 h in the presence of rapamycin.

## Data Availability

The data underlying this article will be shared on reasonable request to the corresponding author.
