# Supplementary material for: Pharmacological inhibition of the PI3K/PTEN/Akt and mTOR signalling pathways limits follicle activation induced by ovarian cryopreservation and in vitro culture
Source: J Ovarian Res. 2021 Jul 19;14:95. doi: 10.1186/s13048-021-00846-5 (PMC8287691; doi:10.1186/s13048-021-00846-5)
Supplement: Supplementary file 1 — Additional file 1. Representative images of LIM-homeobox protein 8 (Lhx8) immunostaining. Ovaries from Nu/Nu mice, 4 weeks old, (A) cultured for 24 h in control medium or (B) cultured for 24 h in the presence of rapamycin. [file 13048_2021_846_MOESM1_ESM.docx]

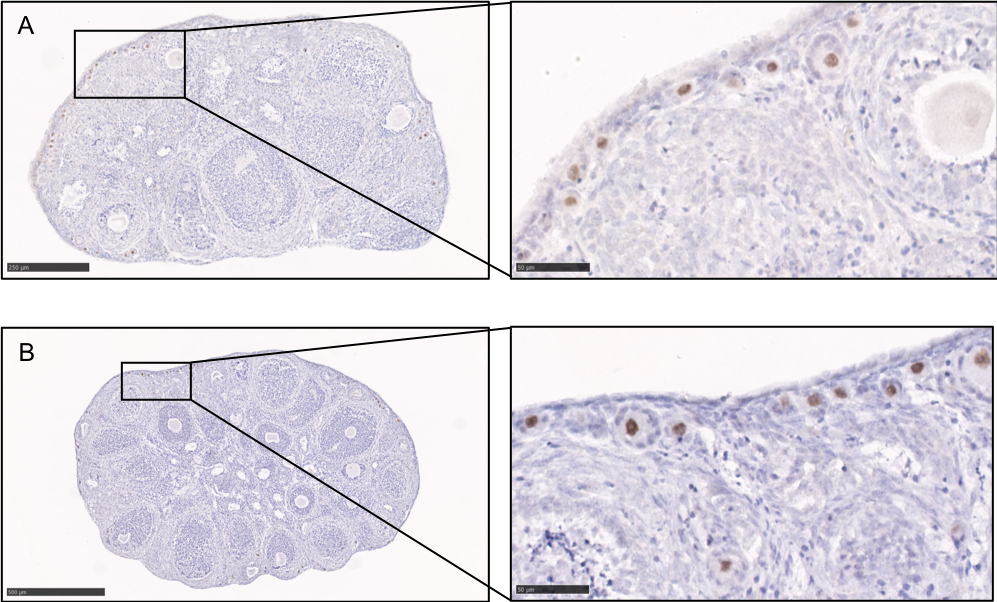


Additional file 1: Representative images of LIM-homeobox protein 8 (Lhx8) immunostaining. Ovaries from Nu/Nu mice, 4 weeks old, (A) cultured for 24 h in control medium or (B) cultured for 24h in the presence of rapamycin.
